# Uniparental disomy: expanding the clinical and molecular phenotypes of whole chromosomes

**DOI:** 10.3389/fgene.2023.1232059

**Published:** 2023-10-04

**Authors:** Qi Chen, Yunpeng Chen, Lin Shi, Ying Tao, Xiaoguang Li, Xiaolan Zhu, Yan Yang, Wenlin Xu

**Affiliations:** ^1^ Genetic and Prenatal Diagnosis Center, Fourth Affiliated Hospital of Jiangsu University, Zhenjiang, China; ^2^ Department of Ultrasound, Fourth Affiliated Hospital of Jiangsu University, Zhenjiang, China; ^3^ Reproductive Medicine Center, Fourth Affiliated Hospital of Jiangsu University, Zhenjiang, China; ^4^ Center for Gynecology and Obstetrics, Fourth Affiliated Hospital of Jiangsu University, Zhenjiang, China

**Keywords:** uniparental disomy, chromosmal microarray analysis, whole exome sequencing, chromosome aberration, phenotype

## Abstract

Uniparental disomy (UPD) refers to as both homologous chromosomes inherited from only one parent without identical copies from the other parent. Studies on clinical phenotypes in UPDs are usually focused on the documented UPD 6, 7, 11, 14, 15, and 20, which directly lead to imprinting disorders. This study describes clinical phenotypes and genetic findings of three patients with UPD 2, 9, and 14, respectively. Chromosomal microarray (CMA), UPDtool, methylation-specific multiplex ligation-dependent probe amplification (MS-MLPA) and whole-exome sequencing (WES) analysis were performed to characterize the genetic etiology. The CMA revealed a homozygous region involving the whole chromosome 2 and 9, a partial region of homozygosity in chromosome 14. UPD-tool revealed a paternal origin of the UPD2. MS-MLPA showed hypomethylation of imprinting gene MEG3 from maternal origin in the UPD14 case. In addition, UPD14 case displayed complex symptoms including growth failure, hypotonia and acute respiratory distress syndrome (ARDS), accompanied by several gene mutations with heterozygous genotype by WES analysis. Furthermore, we reviewed the documented UPDs and summarized the clinical characteristics and prognosis. This study highlighted the importance to confirm the diagnosis and origin of UPD using genetic testing. Therefore, it is suggested that expanding of the detailed phenotypes and genotypes provide effective guidance for molecule testing and genetic counseling, and promote further biological investigation to the underlying mechanisms of imprinted disorders and accompanied copy number variations.

## Introduction

Uniparental disomy (UPD) refers to the inheritance of two homologous chromosomes from one parent (paternal or maternal) without the contribution of identical copies from the other parent (Engel, 1980; Benn, 2021; Gonzales et al., 2022). It is mainly divided into isodisomy UPD (isoUPD), defined as the duplication of a single chromosome inherited from one parent, and heterodisomy UPD (hetUPD), defined as a pair of homologous chromosomes from the contributing parent (Chien et al., 2022). The most frequent mechanism of UPD is caused by nondisjunction events occurring during meiosis and mitosis. Errors in meiosis I or II may result in trisomy or monosomy rescue to correct the aneuploidy. In detail, nondisjunction errors in meiotic I result in the presence of two different homologs from one-single parent or heterodisomy, while errors in meiotic II only result in isodisomy due to the separation error of sister chromatids (Benn, 2021). Furthermore, nondisjunction errors in mitosis result in aneuploidy correction by either trisomy or monosomy rescue. In addition, other rare mechanisms have been identified, including chromosome recombination, gamete complementation and the formation of small supernumerary marker chromosomes (sSMC). Recombination occurring after nondisjunction error in meiotic I or II leads to partial heterodisomy or isodisomy. Gamete complementation refers to an erroneous gamete which is matched with another gamete by a complementary imbalance (Nakka et al., 2019; Del Gaudio et al., 2020).

Unlike single whole-chromosomal UPD, segmental UPD contains only a part of two homologous chromosomes, which may be the result of postzygotic somatic recombination or related to chromosome aberration (Chien et al., 2022). However, there are still particular exceptions where the homoallelic regions start from segmental loss of heterozygosity or consanguinity. It is worth noting that low-ratio or undetectable mosaicism may exist in a significant quantity of UPD cases due to the diverse formation mechanisms of UPD(Eggermann et al., 2015). The clinical outcomes of mosaic chromosome aberration accompanied by UPDs depend on the covered genes and the involved chromosomes. Thus, complete information, including clinical phenotype and ultrasonic examination, will provide useful guidance for genetic evaluation, treatment and prognosis in prenatal.

A recent study revealed that 22 of 5,063 fetal samples had a region of homozygosity, of which five cases were diagnosed with UPDs, with a rate of ∼1/1,000 (Liang et al., 2022). In our center from year 2021–2023, over 1,000 patients underwent amniocentesis, of which three patients were diagnosed with UPD at a rate of ∼0.25%. At present, almost 5148 UPD cases are reported in the database and literature, and the published UPD cases and their phenotypes were summarized and freely available in the online database: https://cs-tl.de/DB/CA/UPD/0-Start.html. Most UPD cases are indicated to have no obvious pathogenic phenotype (Del Gaudio et al., 2020). The abnormal phenotypes mainly result from imprinting gene disorders, autosomal recessive (AR) gene mutations, or the accompanied aneuploidy cells (Gonzales et al., 2022). In general, genomic imprinting involves the DNA methylation of imprinted genes on specific chromosomes and presents differential expression level depending on the parental origin. To date, the definite UPDs associated with the documented imprinted genes include chromosomes 6, 7, 11, 14, 15, and 20 (Chien et al., 2022). The imprinted genes located in the specific regions of these chromosomes will have effects on fetal development, which is possibly resulted from different levels of genetic changes (Miozzo and Simoni, 2002). For instance, UPD14 is caused by a common imprinting disorder of the 14q32 region. Imprinted genes are generally located in this region and regulated by imprinting control regions (ICRs). The imprinting locus contains three methylated regions (IG-DMR, MEG3-DMR, MEG8-DMR), several protein-coding genes (DLK1, RTL1, DIO3), lncRNAs (MEG3, MEG8, RTL1as, DIO3OS) and short ncRNAs (SNORDs and miRNAs) (Rosenfeld et al., 2015). Deletion or abnormal methylation of ICRs leads to imprinting disorder in this cluster. However, these imprinting genes are transcribed based on parental-of-origin, such as protein-coding genes (DLK1, RTL1, DIO3) are paternally expressed, and lncRNAs (MEG3, MEG8, RTL1as) are maternally expressed. Different phenotypes originate from paternal and maternal origin (Garza-Mayén et al., 2021). Kagami-Ogata syndrome (KOS) and Temple Syndrome (TS) are two imprinting disorders in the chromosome 14q32. Specifically, KOS mainly originates from patUPD(14) or epigenetic mutations or deletions on the maternal chromosome region, whereas TS originates from matUPD(14) or epigenetic mutations or deletions on the paternal chromosome region (Briggs et al., 2016; Prasasya et al., 2020).

Herein, we describe data of the three patients with UPD involving in chromosomes 2, 9, and 14, including chromosmal microarray analysis (CMA), and/or whole-exome sequencing (WES) analyses, ultrasonic measurements and clinical outcomes. The CMA results displayed a whole chromosomal region of isodisomy UPD2 and UPD9, but a partial region of homozygosity on chromosome 14. After fully consideration of the loss of heterozygosity (LOH) areas, mosaic regions, and gene mutations, our findings suggested the family single nucleotide polymorphism (SNP) and WES analysis appear to be effective tools for UPD analysis. Together with a briefly review of the documented UPDs, it is suggested that detailed phenotypes and genotypes provide effective guidance for molecule testing and genetic counseling, and promote further biological investigation to the underlying mechanisms of imprinted disorders and accompanied copy number variations.

## Methods

### Design and information collection

We carried out an observational study in fetuses diagnosed with UPDs after amniocentesis. The pregnant women visited our center for genetic counseling between year 2021 and 2023. Medical and family history was collected after a detailed interview. The chromosomal abnormality of fetus was diagnosed by CMA using amniotic fluid through invasive prenatal test. We followed up the ultrasonic measurements during the whole trimester. The normally phenotypic babies were proceeded to observe and follow-up after birth. The trio-SNP, MS-MLPA and WES analysis was recommended after comprehensive counseling.

### Patients and prenatal phenotypes

**CASE 1:** A 31-year-old healthy woman (gravida 1, induced abortion 0, missed abortion 0, para 1) was referred to our center for genetic counseling due to fetal cerebral ventriculomegaly (left side, 10.4 mm). The fetus was conceived through *in vitro* fertilization and embryo transplantation (IVF-ET). In the early trimester of pregnancy, the nuchal translucency (NT) was within normal value (1.8 mm), and the risk of trisomy 21, 18, and 13 using noninvasive prenatal testing (NIPT) displayed a low risk-level. Subsequent amniocentesis was arranged. The couple both denied consanguinity and family history of congenital anomalies. The pregnant woman denied exposure to drugs or radiation during pregnancy.

**CASE 2:** A 31-year-old healthy woman (gravida 1, induced abortion 0, missed abortion 0, para 1) was referred to our center for genetic counseling. She was pregnant with subclinical hypothyroidism. At 18^+3^ weeks of gestation, NIPT showed a high risk of duplication on chromosome 9. Subsequent amniocentesis was arranged. The fetus showed no structural malformation by ultrasound during the whole pregnancy. The parents declared no consanguinity and unremarkable family history, and they decided to continue with the pregnancy after genetic counseling.

**CASE 3:** A 29-year-old healthy woman (gravida 0, induced abortion 0, missed abortion 2, para 0) was referred to our center due to fetal strephenopodia, renal pelvic dilation (left: 0.8 cm/right: 0.53 cm), and right pleural effusion (3.5 mm) (pregnant 24^+2^ week) by ultrasound examination. The NIPT in the early trimester showed a low-risk of Trisomy 21, 18, and 13. The parents declared no consanguinity and unremarkable family history.

### Chromosmal microarray analysis (CMA)

The DNA samples were extracted from amniotic fluid and blood samples using TIANGEN microDNA Kit (TIANGEN, Beijing, China). Briefly, DNA was digested, ligated to adaptors, and amplified by PCR. Then, purified DNA was fragmented, biotin-labeled and hybridized to the 750K chip. Chromosome Analysis Suite (ChAS) software (Affymetrix, Santa Clara, CA) was used to analyze the raw data and visualize the results based on the GRCh38 assembly.

### UPDtool analysis

UPDtool_0.2 is used to detect and classify UPD origin in trio-SNP-microarray experiments. The family microarray data was analyzed with ChAS software. The family genotype information allows detection of inheritance errors, called Mendelian errors (MEs). These data contains SNP markers of same inheritance that can be both informative (i.e.,.MEs) and non-informative. The genotype data was exported and converted to the genotype (GT) input format files using UPD converter tool. Then the GT file was rearranged in the order of chromosome, location, father’s genotype, mother’s genotype, and proband’s genotype (Schroeder et al., 2013). The chromosome and location columns were moved to the beginning of the spreadsheet. UPD-tool_0.2 software was used to analyze these data.

### Methylation-specific multiplex ligation dependent probe amplification (MS-MLPA)

MS-MLPA analysis was performed using a SALSA MS-MLPA kit (ME032, MRC Holland, Amsterdam, Netherlands) according to the manufacturer’s instruction. The specific probes were hybridized to the denatured DNA, then one sample was directly ligated, the other one was digested with the HhaI methylated-specific restriction enzyme before ligation. PCR was performed using fluorescence-labeled unique primers for probe sets provided in the SALSA MLPA kit. The PCR products were resolved on an ABI Prism 3730 Genetic Analyzer (Applied Biosystems, CA, United States) by Coffalyser software (http://www.coffalyser.net).

## Results

### Genetic diagnosis

**FIGURE 1:**
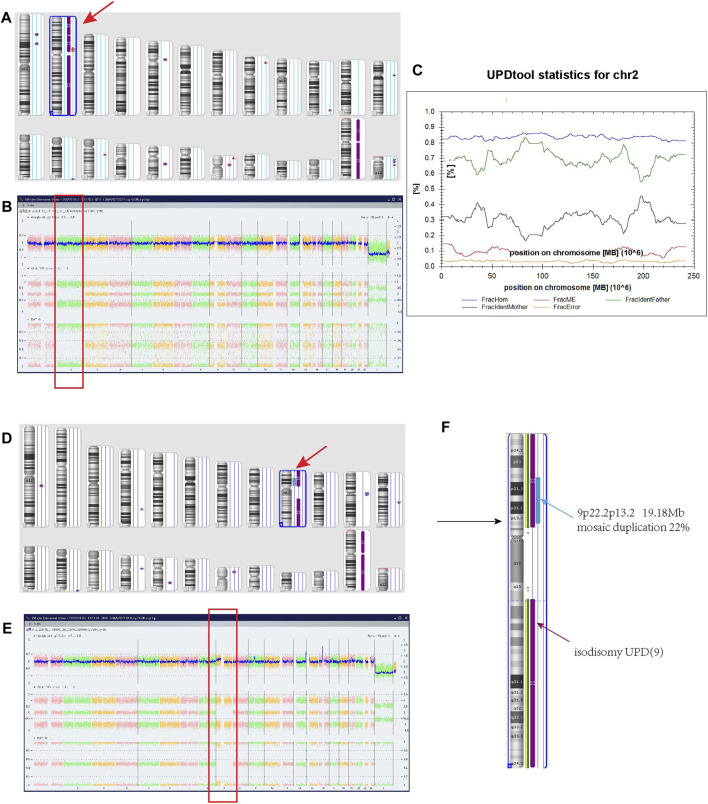
Regions of homozygosity (ROH) in chromosomes 2 and 9 identified with SNP array analysis of the fetus and UPDtool analysis showed a complete paternal uniparental isodisomy (isoUPD) of chromosome 2. **(A)** ChAS revealed a complete ROH across the entire chromosome 2 (purple rectangle, red arrow). **(B)** A whole chromosome view clearly shows the copy neutral ROH on chromosome 2 in the fetus (red box). **(C)** Classification of UPD using the UPDtool showed the fetus was complete paternal isoUPD. FracHom (blue line) is the fraction of homozygous SNPs, FracME (red line) is the fraction of Mendelian error SNPs, FracldentFather (green line) is the fraction of SNPs where the genotype is identical to the father, FracldentMother (black line) is the fraction of SNPs where the genotype is identical to the mother, and FracError (yellow line) is the fraction of errors. **(D)** ChAS revealed a complete ROH across the entire chromosome 9 (purple rectangle, red arrow). **(E)** A whole chromosome view clearly shows the copy neutral ROH (isodisomy area) on chromosome 9 in the fetus (red box). **(F)** A partial gain region of 9p22.2p13.1 with 22% mosaic duplication (blue arrow).

For case 1, The SNP array using uncultured amniocytes did not reveal any pathogenic copy number variants. However, the result suggested a region of homozygosity across the entire chromosome 2 (Figures 1A, B). Further, the trios-SNP microarray data showed the fetus had a complete paternal isoUPD(2) by UPD-tool statistics (Figure 1C).

For case 2, the SNP array using uncultured amniocytes revealed a complete homozygous region across the entire chromosome 9 (Figures 1D, E), and accompanied by a gain mosaic region in the 9p22.2p13.2 (chr9:17986289–37173112) with a ratio of 22%, reaching the size of 19.18 Mb (Figure 1F). These findings suggested the existence of isodisomy UPD and partial duplication of interstitial 9p. For this duplication area, the Clinical Genome Resource (ClinGen) and Decipher database showed that it encompassed 132 protein coding genes, but no definite clinical phenotypes were reported in the recent studies. Based on the standard of the American College of Medical Genetics and Genomics (ACMG), although this area is accompanied by a low mosaic ratio, it is still considered likely pathogenic.

**FIGURE 2:**
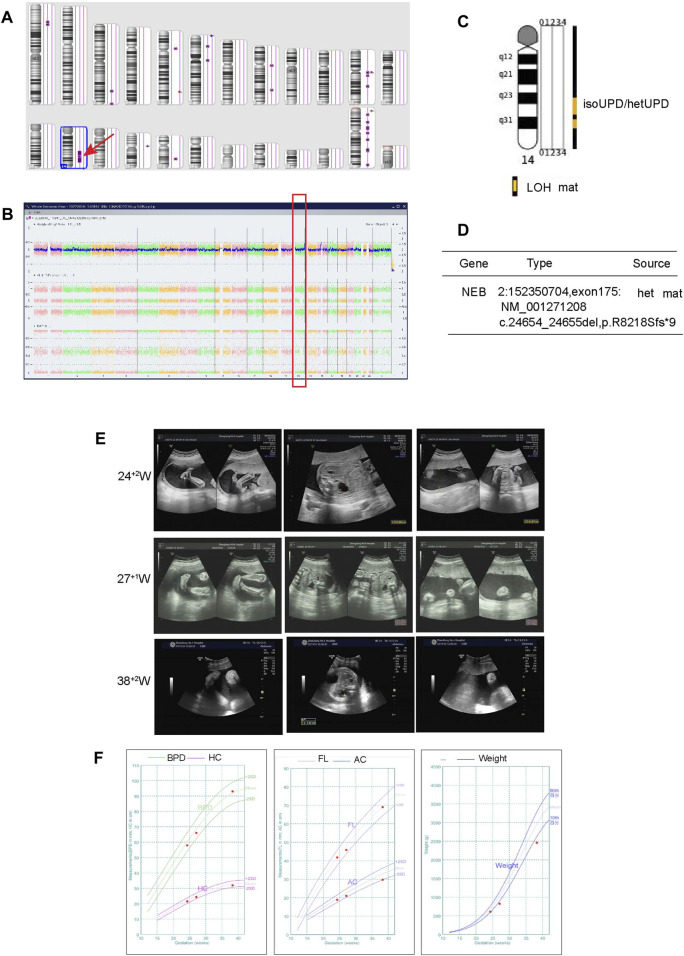
Regions of homozygosity (ROH) in chromosome 14 identified with SNP array analysis of the fetus and trio-WES analysis showed a maternal uniparental isodisomy (isoUPD) and uniparental heterodisomy (hetUPD) of chromosome 14. **(A)** ChAS revealed a partial ROH in the long arm area of chromosome 14 (purple rectangle, red arrow). **(B)** A whole chromosome view clearly shows the copy neutral ROH on chromosome 14 in the fetus (red box). **(C)** A trio-WES analysis showed that a complete maternal mixed-UPD of chromosome 14. **(D)** The trio-WES analysis showed that NEB gene mutation from maternal origin. The prenatal phenotypes of the fetus by ultrasound and the fetal growth curve in different gestational week. **(E)** The ultrasound examination demonstrated the existence of strephenopodia, renal pelvic dilation at 24 + 2 weeks, left hydronephrosis and polyhydramnios at 27 + 1 and 38 + 2 week. **(F)** The growth curve including BPD, HC, FL, AC, estimate weight in 24 + 2, 27 + 1 and 38+2 weeks, respectively.

For case 3, the SNP array showed a LOH region on the 14q23.2q32.12 [(62065219–91904797) x2 hmz] (Figures 2A, B), without any other copy number variations (CNVs). Based on the ClinGen database, there are 172 protein coding genes involving in this homozygous region. However, the documented imprinted genes, either maternal expressed (e.g., MEG3, RTL1as, and MEG8) or paternally expressed (e.g., DLK1 and RTL1), are localized outside. The LOH region includes only 7 Online Mendelian Inheritance in Man (OMIM) genes out of the 172 coding genes are related to AR genetic pattern with definite evidence. In Supplementary Table S2, a summary of their detailed genotype, phenotypes in previous reported individuals with homozygous mutation are presented. Surprisingly, it is revealed that homozygous variants of POMT2 is related to muscular dystrophy, displaying hypotonia, low left ventricular ejection and mild restrictive lung disease. Except for POMT2, the other diseases due to the mutated variants have not been reported in the UPD14 patients yet. And the phenotypes of homozygosity of these gene mutations are not in consistent with our case. The WES results did not find these mutations in our patient (Supplementary Table S1), but demonstrated a mixed iso- and hetero-disomy (iUPD/hUPD)14 from maternal origin (Figure 2C). Moreover, it was noticed that the baby inherited a maternally heterozygous NEB mutation (2q23, c.24654_24655del) and a POLRMT mutation (c.1016T>C,p.L339P) (Figure 2D; Supplementary Table S1). In detail, the NEB mutation may cause Nemaline myopathy, and POLRMT is a key enzyme for transcription of the mitochondrial genome. We found that partial of the clinical phenotypes of these two gene mutations including mild development delay, hypotonia, short statue are consistent with our patients. Although the NEB variant is not considered the pathogenic cause with only heterozygous mutation, it is noting that the POLRMT variant may act as recessive or dominate inheritance. In addition, MS-MLPA analysis displayed normal copy number changes with a peak ratio value ∼1.0 (two copies) at the 14q32 region (Supplementary Figure S1). The methylation ratio at MEG3 locus was ∼0 in comparison with the ∼0.5 methylation ratio from a normal control, suggesting that the fetus was TS patient with a paternal allele deletion.

### Clinical manifestation and outcome

For UPD(2) fetus, head magnetic resonance imaging (MRI) in the third trimester indicated that the fetal craniocerebral structure is normal. The fetus presented normal phenotype during the whole pregnancy and the width of lateral ventricle is within the normal range of 9 mm in the third trimester of pregnancy. At 38^+3^ week of gestation, a 3,600 g male-infant was delivered by caesarean and had no abnormal phenotype. Among the four UPDs from recent literature, two of them displayed normal phenotype. The other two cases exhibit unspecific phenotypes, including a mild intellectual disability, IUGR, growth failure, multiple minor malformations. The karyotypes are normal, but accompanied by either confined placental mosaicism or different gene mutations (FAM16A, NAT8, PLA2R1) (Table 2). Furthermore, a summary of clinical features in the isodisomy patUPD2 patients in previous reported individuals with homozygous mutation are presented in Supplementary Table S3.

For UPD(9) case, the pregnant woman had a caesarean at 37^+4^ weeks of gestation, and a 3,130 g male infant was delivered with normal physical findings (Apgar score = 10). At the aged of 9 months, the baby had achieved his appropriate developmental milestones by physical examination and presented no physical abnormalities except cryptorchidism. Otherwise, the four UPD9 cases from the literature showed a different ratio of T9 mosaicism or sSMC in Table 2. After confirmation by blood testing, two of the true T9 mosaicism neonatal baby showed different phenotypes, including feeding problem, minor facial dysmorphism, even accompanied by intellectual disability. However, patients with T9 placental mosacism or sSMC karyotype displayed normal phenotypes. Furthermore, a summary of clinical features in the UPD9 patients in previous reported individuals with homozygous mutation are presented in Supplementary Table S3.

For UPD(14) fetus, the ultrasonic examination showed a urinary tract dilation (left side, from1.36 cm at 27^+1^w to 1.4 cm at 38^+2^w), strephenopodia and polyhydramnios (AFI: 31.4 cm at 38^+2^w) (Figure 2E). The estimated fetal weight (EFW) was located at 2.6% of the average EFW. In Figure 2F and Table 1, the fetal growth curve and parameters showed that except for the weight exhibited a gradual loss trend (<-2SD in the third trimester), the others measurements were generally in accordance with gestational age. Finally, a female baby was delivered at 39^+4^ weeks. There was no obvious obstruction in the bladder and ureter after birth. The neonatal baby is small for gestational age with a birth weight of 2.2 kg (−3.54 SD), accompanied with hypotonia, irregular and wheezy breathing, skin cyanosis, and quickly died of acute respiratory distress syndrome (ARDS) and shock. After careful physical examination, the infant exhibited strephenopodia, hypotonia, scattered bleeding points in the front chest and lower limbs, two masses on both sides of the head (1 cm × 1 cm), which was beyond the cranial suture without wave sensation. There were no deformities in the spine and limbs, but all the fingers were flexed and clawed-like, and could not be straightened. The second knuckle of the index finger, middle finger and index finger has no transverse lines. The ultrasonic examination suggested normal cranial structure and renal collecting system, but the size of both kidneys was larger than normal (left: 44 × 18 mm, right:43 × 16 mm) with left-side urinary tract dilation (8 mm); normal cardiac function but atrial septal defect (5.3 mm), patent ductus arteriosus, pulmonary hypertension (systolic pressure 45 mmHg), moderate tricuspid regurgitation, mild mitral regurgitation. Based on the maternal UPD14 patients, nearly all the patients showed intrauterine growth retardation (IUGR), neonatal hypotonia and small hands/feet. The other symptoms are including joint hypermobility, precocious puberty, small for gestational age (SGA), tube feeding after birth and psychobehavioral problems, etc., (Table 2).

**TABLE 1:** Ultrasonic parameters for UPD(14) patient in correspondence with different gestational ages.

Gestational Age(W)	BPD (cm)	HC (cm)	AC (cm)	FL (cm)	HL (cm)	Weight (g)	Placental thickness (cm)	Amniotic fluid volume/index (cm)	Prenatal ultrasound phenotypes
24^+2^	5.79	21.5	18.8	4.18	3.82	608 (14%)	2.39	4.47 (AFV)	Strephenopodia
									Urinary tract dilation (left 0.8/right 0.53 cm)
									Right pleural effusion (3.5 mm)
27^+1^	6.6	24.3	21	4.58	4.1	824 (1.5%)	2.98	6.33 (AFV)	Strephenopodia, slightly retracted lower jawbone
									Urinary tract dilation (left 1.36 cm)
38^+2^	9.3	31.9	29.7 (2.3%)	6.9	—	2457 (2.6%)	3.8	31.4 (AFI)	Strephenopodia
									Enlarged size of left kidney
									Urinary tract dilation (left 1.4 cm)

BPD, biparietal diameter; HC, head circumference; FL, femur length; HL, humerus length; AC, abdominal circumference; W, week.

**TABLE 2:** Summary of clinical phenotypes in the whole chromosome UPD(2) and UPD(9) cases.

	Origin	Imprinted genes/Gene mutation	Amniocyte (prenatal)	Blood testing (neonatal/child)	Diseases	Phenotypes	PMID
UPD 2							
Zhang et al. (2019)	Pat	No/No	—	WGS and STR: completely pat UPD2	—	Normal phenotype	30991391
Song et al. (2021)	Unknown	No/No	UPD 2	46,XX.	—	Normal phenotype	33678350
Carmichael et al. (2013)	Mat	No/FAM161A, NAT8, PLA2R1	UPD 2	46,XX.	skeletal and renal dysplasia, immune deficiencies, growth failure, retinal degeneration and ovarian insufficiency	Severe IUGR (26w gestation), low birth weight (-3.6SD), mild global developmental delay, brachydactyly, pes planus	23167750
Hansen et al. (1997)	Mat	No/No	Placental biopsy: T2	—	—	IUGR and oligohydramnios (24 weeks’ gestation), hypospadias	9178319
			Karyotype: 46,XY				
			STR:UPD 2				
UPD 9							
Slater HR et al. (2000)	Mat	—	Karyotype:47,XX,+9[2]/46,XX[69]	4/50 mosaic trisomy 9	—	feeding and growth problem after birth; minor facial dysmorphism (small posteriorly rotated ears and thickened helices, micrognathia and a long, narrow face) and skeletal abnormalities	11113900
			STR:UPD 9				
Ma et al. (2015)	Pat	—	UPD9/T9 mosaic (20%)	Isodiosomy paternal UPD9/T9 mosaic 20%	—	severe motor and intellectual disability, recurrent respiratory infection and failure to thrive. Severe growth retardation, moderate to severe hypertonia. Dysmorphic features	26120364
Chen et al. (2022)	Mat	—	47,XX,+9[4]/46,XX[35]	Karyotype: 46,XX.	IUGR, Preeclampsia	Normal phenotype (6 months baby)	35181026
			UPD9 mat/mosaic T9 (14%)	Placenta: maternal T9			
			FISH: mosaic T9 10.7%	Postnatal FISH: no T9			
Chen et al. (2017)	Pat		47,XY,+mar [25]/48,XY,+mar,+r(9) [4]/47,XY,+r(9) [1]/46, XY [6] 9p13.1q21.11 (38,792,472-71,026,063)x2.64 *de novo*	47,XY,+sSMC(9) [14]/48,XY, +sSMC(9),+r(9) [10]/47,XY,+r(9) [6]/46, XY [10]	—	Normal phenotype, psychomotor and growth development	28805612
				9p22.3q21.11(14234165–71035608)×2-3			
				9p24.3p22.3(216,123-14,629,321)x2 hmz			
				9p21.3p13.2(24769722-36732597)x2 hmz, and 9q21.11q34.3(71013799-141011581)x2 hmz			

## Discussion

With the widespread application of genetic technologies, increasing UPD cases involving different chromosomes have been detected. In addition to pathogenic UPDs, other chromosome UPDs are still considered uncertain variants and are usually followed up with clinical observations. In this study, we described a complete isoUPD2, an isoUPD9 and a mixed UPD14 case in prenatal after amniocentesis. The prenatal ultrasound showed no structural abnormalities and a normal growth rate during the whole pregnancy in the UPD2 and UPD9 cases. The two neonates displayed normal phenotypes, and the 9-month-old baby with UPD9 showed normal growth and development so far. Unfortunately, the UPD14 case developed with polyhydramnios, hydronephrosis and low EFW in late pregnancy. Hypotonia and respiratory dysfunction progressed rapidly after birth, and the newborn died of cardiopulmonary failure. Together, these cases provide new references that UPDs across different chromosomes may result in a spectrum of normal to severe phenotypes in the neonates; moreover, not all the phenotypes can be discovered in prenatal. It is suggested that effective testing methods and comprehensive evaluation of the detailed phenotypes during genetic counseling are considered appropriate strategies and directions for UPD patients.

Generally, the primary mechanisms of isoUPD may be generated from trisomy or monosomy through meiotic and mitotic errors (Engel, 1980; Kearney et al., 2011). In surviving zygotes, the trisomy has been removed entirely; alternatively, structural reduction of trisomic chromosome or conversion to a marker or ring chromosome may occur, or the mosaic status may exist. These processes are collectively called trisomy rescue, which is the most frequently cause of the UPDs. Specifically, depending on the origin and random arrangement of recombination, UPDs can be completely isodisomic, heterodisomic, or mixed iso- and heterodisomic (Eggermann et al., 2015). In the same situation, zygotes can be rescued only by duplication of the monosomic chromosome, thereby resulting in whole-chromosomal isoUPD(Benn, 2021).

Based on the UPD database and recent literature, clinical manifestation of UPDs with unbalanced karyotype or CNVs, or mosaic cells, was summarized in Table 2 and Supplementary Table S3. It is noting that most of UPD2 and UPD9, either maternal or paternal source, displayed normal or balanced karyotype without clinical findings, including segmental UPD. In fact, the phenotypes are definitely associated with origin and the certain chromosome, which are mainly resulted from the imprinted dysregulation, or AR diseases, or the presence of mosaic cells. In this study, we aimed to display different clinical features between imprinting and non-imprinting UPDs. Besides our patUPD2 sample, several studies have showed that UPD2 patients exhibit normal phenotypes (Keller et al., 2009; Zhang et al., 2019; Song et al., 2021). However, rare matUPD2 patients accompanied with unspecific phenotypes, including severe IUGR, mild development delay (Ou et al., 2013; Zhou et al., 2017), oligohydramios and hypospadias (Hansen et al., 1997) (Table 2). Furthermore, special cases in either mat or patUPD9, accompanied with different mosaic level of trisomy 9, or marker chromosomes, displayed multiple phenotypes, from normal to severe (Chen et al., 2017; Chen et al., 2022), including minor dysmorphism, skeletal abnormalities (Slater et al., 2000), intellectual disability, and growth retardation (Ma et al., 2015) (Table 2). A special case of matUPD(9) was noted with purulent chorioamnionitis and retarded embryo growth, resulting in spontaneous abortions (Slater et al., 2000). In this way, we must admit that our UPD2 and UPD9 cases have not provided novel clinical information. However, these cases are still important proofs that chromosome 2 and 9 are not subjected to imprinted gene disorders. The precious normal phenotypes provided useful references for clinical outcomes of these two UPDs in prenatal. Certainly, the follow-up procedure will be continued and collected, including growth development, language, motor, and other phenotypes, which are important guidance to provide further treatments and prognostic evaluations.

Genomic imprinting is an epigenetic regulation and is closely associated with the pathogenicity of UPDs (Soellner et al., 2017). Many studies have demonstrated that disruption of imprinted genes is relevant to phenotypic changes associated with retarded growth and development (prenatal or postnatal) (Miozzo and Simoni, 2002; Soellner et al., 2017), hypo-/hyperglycemia, abnormal feeding behavior, intellectual disability and precocious puberty (Soellner et al., 2017). In this study, we described a special case of mixed-UPD14 diagnosed with TS, accompanied by severe clinical characteristics, the neonate quickly died of severe ARDS. Mechanically, TS arises from a maternal UPD14 (65%–70%), paternal deletion of 14q32 (5%–15%), or epimutation at the IG-DMR (10%–20%), with/without a robertsonian translocation of chromosome 14 (Prasasya et al., 2020). In our case, the LOH region is located at 14q23.2q32.12, and the 14q32 imprinting locus is partially contained within the isodisomic region. The mixed large area of segmental isoUPD and heteroUPD may be related to meiotic crossing-over of maternal cells, followed by trisomy rescue (Benn, 2021). Additionally, MS-MLPA result displayed a hypomethylation of MEG3. In particular, the imprinted SMOC1, MEG3/8, and SNORD113-1/114-1 genes were maternally expressed (Sabria-Back et al., 2022). The diagnosis of TS can be definitely identified, caused by methylation defects of MEG3 hypomethylation. However, TS may not be the only explanation for the complex phenotypes in this case. Thus, we infer an existence of the other methylated disorders of the DMRs region from maternal origin, which can lead to functional disruption of the imprinted gene expression. It is worth noting that two genes, SMOC1 and ESR2, are imprinted genes located in the 14q24.2 and 14q23.2-q23.3 regions (https://www.geneimprint.com/site/genes-by-species). In addition, premature rupture of membranes occurred before fully-opened of the uterine, and transient deceleration of fetal heart rate (down to 80 beats per minute) was observed during the labor. Although it is insufficient evidence for intrauterine distress, neonate was intubated and ventilated with positive pressure for severe asphyxia after birth. We hypothesized that premature rupture of membranes and fetal heart rate deceleration during the labor may related to the intrauterine distress, which may be one of the possible reasons for fetal respiratory dysfunction.

To date, clinical phenotypes of TS have been revealed and elucidated. Based on the complicated mechanisms of UPD14, other nonspecific phenotypes still need to be exploited (Prasasya et al., 2020). The typical characteristics of TS mainly included low birth weight (caused by intrauterine growth restriction in antenatal), hypotonia (mainly related to poor feeding and limited suck reflex after birth) (Kagami et al., 2017b), motor delay, mild facial dysmorphism (a broad forehead, short nose with a wide nasal tip, or small hands/feet), feeding problems, short stature and premature puberty (Ioannides et al., 2014; Sabria-Back et al., 2022). Obviously, growth failure and hypotonia for our UPD14 patients are consistent with the typical phenotypes of TS. Unfortunately, severe dyspnea is a kind of exception. In the reported isolated TS, nearly all the patients showed IUGR, hypotonia and small hands/feet, other phenotypes included joint hypermobility, precocious puberty, SGA, tube feeding after birth and psychobehavioral problems (Chan et al., 2019; Juriaans et al., 2022). For instance, a 4-year-old patient with hetero- and isoUPD14 from mothers on chromosome 14q11.2q24.3 (14q11.2q24.3 (20520197–76786044) x2 hmz) exhibited low birth weight, hypotonia, motor retardation, feeding problems and short stature (Shin et al., 2016). In addition, only a few patients with TS demonstrated obesity, type 2 diabetes mellitus, inguinal hernia, constipation, hyperparathyroidism (Chan et al., 2019) and cognitive development from normal to moderately delayed (Juriaans et al., 2022). So far, TS demonstrated a milder condition, and there is no reported case the same as ours that results in such a rapid death of severe respiratory failure and shock.

Previous studies have demonstrated that KOS caused by patUPD14 exhibited unique phenotypes, including thoracic skeletal anomalies, polyhydramnios, placentomegaly and growth failure (Kagami et al., 2017a). Notably, KOS infants may exhibit a small bell-shaped thorax, coat-hanger ribs and narrow chest wall, leading to significant respiratory distress after birth. Moreover, they often require intubation and intensive care with oxygen and respiratory monitoring systems (Prasasya et al., 2020). Some of them also exhibit mild craniofacial deformities, short neck, short palpebral fissures, anteverted nares and micrognathia (Kagami et al., 2015). Polyhydramnios is common and newborns often exhibit macrosomia in KOS(Kagami et al., 2015; Prasasya et al., 2020), while oligohydramnios and small placenta exist in TS. Although it is difficult to summarize all the phenotypes of TS, especially those cases result from methylated dysfunction (Briggs et al., 2016), it can be seen that these clinical features are partially consistent with our UPD14 case, those severe phenotypes indicate that there exist more etiologies to be identified in addition to the diagnosis of TS.

As we known, the documented 14q32.2 imprinted region is characterized by three DMRs and a cluster of imprinted genes. The clinical phenotype is caused by disruption of this region with unbalanced imprinted gene expression. In fact, it is not yet determined if the different etiologies cause identical phenotypes or the pathogenic area is only attributed to the 14q32.2 region. Firstly, the clinical phenotypes of isoUPD may possibly result from rare AR disorders. For instance, an 11-month-old girl was diagnosed with matUPD14 and a homozygous mutation of the SLC7A7 gene located 14q11.2, leading to lysinuric protein intolerance (LPI) (Kang et al., 2019). In our UPD14 case, the family members denied the relevant genetic history, and all the family members have normal phenotype. The two abortion pregnancies were not performed genetic examination. Based on the poor prognosis, we doubted that whether these phenotypes (e.g., ARDS and polyhydramnios) could be explained by certain mutations across the chromosomes. WES result showed a NEB variant (c.24654_24655del, p.R8218Sfs*9, in exon157, het) and a POLRMT variant (c.1016T>C, p.L339P, het). The phenotypes of NEB and POLAMT variants are partial in accordance with myopathy. However, these two variants are usually recessively inherited (Sewry et al., 2019). There is only one dominantly inherited patient, causing a distal form of nemaline myopathy in a three generation family with a large region of deletion (Kiiski et al., 2019). In addition, there is a study reported eight patients with POLRMT mutations associated with mitochondrial dysfunction and neurological disorders. It is noting that two of them are identified heterozygous variants c.2641-1G>C, p.Gly881_Lys883del; c.1832C>T, p.Ser611Phe, the clinical phenotypes including mild development delay, hypotonia, short statue. Thus, we cannot ignore the probability of the POLRMT variant (c.1016T>C,p.L339P) acting as dominant inheritance (Oláhová et al., 2021). In the LOH region, we found a homozygosity of POMT2 variants could result in muscular dystrophy. But our cases did not carry this mutation. Therefore, except TS, we have not found the exact etiology to explain all the phenotypes of our patients yet, but NEB and POLAMT may be the possible risks, which still need to be validated. Unfortunately, in this paper, no definite gene mutation could be considered a pathological cause by WES analysis. Rare regulated functions of genes in this homozygous region may be responsible, or erroneous methylated regions in the other areas of the genome.

Furthermore, CMA result demonstrated a mosaic region of partial 9p duplication with a ratio of 22% in case 2. The duplicated region contained large numbers of protein-coding genes, but there is not yet certain pathogenic evidence from recent literature. In most cases, the partial trisomy of 9p comes from a parent carrying a reciprocal balanced translocation, accompanied by the simultaneous deletion of another chromosome. Patients with typical 9p duplication may exhibit growth/intellectual disability and microbrachycephaly (Leone et al., 2020). There was reported a girl with a duplicated region extending from 9p22.1 to 9p13.1 exhibiting minimal physical findings (Bonaglia et al., 2002). The other pathogenic phenotypes may result from homozygous allele mutations (Nishimura et al., 2020) and different ratios of mosaic duplication (Slater et al., 2000; Ma et al., 2015) in isoUPDs. Otherwise, we reviewed the TS cases and noticed that a mosaic T14 may be accompanied by disomic cells (Ushijima et al., 2018). Since the CMA result was not reliable in detecting mosaicism below 30% and was not used to analyze gene methylation (Lindgren et al., 2021), we assumed that if a low proportion of mosaic T14 likely existed in our UPD14 case. Studies have reported that 10 live UPD14 patients coexisted with mosaic trisomic cells. These patients are believed to have specific phenotypes of T14 and TS (Garza-Mayén et al., 2021; Lindgren et al., 2021). According to these reports, mosaic T14 cases may display phenotypes of frontal bossing, ocular hypertelorism, widening of the posterior cranial fossa, micrognathia, abnormal cardiac structure, strephenopodia in early pregnancy, IUGR, hydramnios/oligohydramnios, cleft palate, high arched jaw, microcephaly, pericardial effusion, cardiac malformation, omphalocele, clenched fist and syndactyly in the late trimester (Massara et al., 2019), some of which are partially consistent with our UPD14 case. However, the final phenotypes are dependent on the percentage of mosaicism ratio and tissue distribution of the mosaic cells.

## Conclusion

We present three patients with UPD (2), (9), and (14), respectively; and reviewed related UPDs from recent literature. The different phenotypes and prognosis of our cases provide important supplements to the existed UPD cases. To date, many studies have demonstrated a favorable outcome for UPD2 and UPD9 samples. However, UPD14 are completely different. The clinical phenotypes of UPD14 patients are diverse, especially when it is accompanied by homozygous mutations or methylated disruptions, leading to complicated symptoms. In general, the prognosis of TS is much better than KOS. Due to the low incidence, clinical characteristics of UPDs involved different chromosomes may be unpredictable, which increases the difficulty of genetic counseling, especially in prenatal. In addition, the potential roles of imprinting disorders have not been fully explored and analyzed. Thus, detailed investigation in prenatal, including cytogenetic analysis, molecular testing (such as CMA, MLPA, MS-MLPA, and WES), as well as ultrasonic measurements may be useful methods and guidance for consultation. The long-term prognosis is not only based on these comprehensive analyses, but also on followed-up observation after birth.

## Data Availability

The original contributions presented in the study are included in the article/Supplementary Material, further inquiries can be directed to the corresponding author.
