# Supplementary material for: Uniparental disomy: expanding the clinical and molecular phenotypes of whole chromosomes
Source: Front Genet. 2023 Oct 4;14:1232059. doi: 10.3389/fgene.2023.1232059 (PMC10582337; doi:10.3389/fgene.2023.1232059)
Supplement: Supplementary file 1 [file DataSheet3.docx]

**Supplementary Table 3**. Summary of clinical phenotypes for UPD cases involved other non-imprinted chromosomes.

| **UPD(chromosome region)**  **parental origin** | **ROH (Mb)** | **Age** | **Results** | **Manifestations** | **PMID** |
| --- | --- | --- | --- | --- | --- |
| UPD(1pter→p36.22)  mat | 9.39 | prenatal | AF:46,XN,der(1)t(1;17)(p36.3;q21)[48]/46,XN[2] PBL at 9y:46,XN | multiple congenital anomalies | 34284807 |
| UPD(1pter→p36.22)  mat | 9.33 | prenatal | NIPT: del(1)(p36) AF: 46,XN | normal sonography | 34284807 |
| UPD(1pter→p36.13)  mat | 16.32 | prenatal | NIPT: del(1)(p36.23) and dup(1)(p36.23p36.22) in child (blood) 80% of cells with imbalance | normal as child | 34284807 |
| UPD(3pter→p22.1)  mat | 43.13 | 24y | at 4 y: 46,XN,der(3)t(3;4)(p22;q35)[40%]/46,XN[60%] at 24y der(3) only in 5% | Developmental delay, obesity, hearing loss | 34284807 |
| UPD(4)  pat | - | prenatal | 46,XN,+4 acc. to NIPT | termination of pregnancy (TOP) | 36226167 |
| UPD(5pter→p15.1)  mat | 16..44 | 51y | n.a. | Cardiomyopathy, hypertension, mental retardation | 34284807 |
| UPD(5)  pat | - | prenatal | 47,XY,+5 in chorion 46,XY in AF and PBL | IUGR; normal at birth At 1 years, central hypotonia, lack of coordination, torticollis, and delay in motor skills | 31251480 |
| UPD(8)  pat | - | prenatal | 46,XX  NIPT: trisomy 8; none prenatally and none at 3y | normal | 32599602 |
| UPD(8p23.1→pter)  pat | - | 12y | aCGH: der(8) with del and dup in 8p23.1 | At birth atrial septal defect(ASD) type II; at 12y moderate intellectual disability(IQ = 50), obesity | 28602932 |
| UPD(12)  mat | - | prenatal | 47,XX,+12/ 47,XX,+i(12)(p10)/ 46,XX  FISH: n.a. | Pallister Killian syndrome, TOP | 15614858 |
| UPD(12)  mat | - | prenatal | 47,XX,+12[2]/46,XX[45] | Advanced maternal age (AMA); sonographic abnormalities, TOP | 8606880 |
| UPD(16)  pat | - | prenatal | 47,XX,+16/ 46,XX | IUGR; mild facial abnormities, VSD, ASD | 8953635, 10756340 |
| UPD(16)  Pat+sSMC | - | prenatal | 47,XY,+mar[27]/ 46,XY[5]  FISH: r(16)(::p11.1→q11.2::) | Dandy Walker Cyst and brainmalformations; TOP | / |
| UPD(16)  Mat | - | prenatal | 46,XN,+16  acc. to NIPT | TOP | 36226167, 35522888 |
| UPD(17q25→qter)  Mat | - | n.a. | 46,XY | severe MR, macrostomia, thick upper lip, hypertelorism, epicanthus, aggressive behavior; | 11950856 |
| UPD(17pter→p13.3)  unknown | 1.91 | 13y | aCGH x3: 17p13.3-17p13.2 | Developmental Delay(DD) | 34284807 |
| UPD(17q25.3→qter)  unknown | 2.23 | 2.5y | aCGH x3: 17q25.3-17q25.3 | DD | 34284807 |
| UPD(17)  unknown | - | prenatal | 46,XN,+17  acc. to NIPT | TOP | 36226167 |
| UPD(18)  Mat+sSMC | - | prenatal | 47,XX,+mar[16]/ 47,XX,+18[2]  **FISH: min(18)(:p11.1→q11.1:)** | Advanced maternal age, no sonographic signs; TOP; no external abnormalities | 30171908 |
| UPD(18)  Pat | - | 3y | n.a. | Delayed speech, overlapping 4th finger | 21594998 |
| UPD(18)  unknown | - | prenatal | **aCGH: del(X)(p21.1),+16,+18(mosaic) 18p11.31 to 18q12.1 and 18q21.31 to 18q23.** | **spontaneous abortion** | 24914406 |
| UPD(18)  unknown | **Size unclear** | n.a. | **47,XY,+18[2]/ 46,XY[7]** | **most likely dysmorphism(DYS) and mental retardation(MR)** | **25118026** |
| UPD(19)  Pat | **-** | newborn | **46,XX** | **DYS,DD** | **30007940** |
| UPD(19)  unknown | **-** | postnatal | **n.a.** | DD, hypotonia (twins) | 31767986 |
| UPD(21)  mat | **-** | Early pregnancy | **46,XN** | Early embryonic failure | 7987317 |
| UPD(21)  mat | **-** | prenatal | **46,XN** | Abnormal sonography | 25863078 |
| UPD(21)  mat | **-** | 5y | **46,XY** | DD; congenital abnormalities of the nervous system | 21594998 |
| UPD(21)  pat | **-** | 8th week of gestation | 46,XX | missed abortion | 11840192 |
| UPD(21)  pat | **-** | newborn | 46,XX in PBL after ~1year initially and in fibroblasts: 46,XX,r(21)/46,XX,-21 | growth failure, facial DYS, muscular hypertonia, and severe psychomotor retardation | 7966190 |
| UPD(21)  pat | **-** | 10m | 45,XX,-21,dup(21)(q22.11q22.3) | short stature, DYS, mild motor delay | 30123325 |
| UPD(22)  unknown | **-** | postnatal | n.a. | Endometrial, thyroid, sigmoid, breast, and kidney cancer | 31767986 |
| UPD(22)  unknown | **-** | postnatal | 46,XN | different neurodev. disorders | 33495530 |
| UPD(22)  mat | **-** | prenatal | n.a. | malformations | 21056321 |
| UPD(22)  mat | **-** | prenatal | 46,XY in PBL 47,XY,+22 in placenta | growth retardation, DYS | 9412788 |
| UPD(X)  Pat | **-** | 28y | 46,XX pat transmission of both X | recurrent miscarriages | 21594998 |
| UPD(X)  Pat | **-** | n.a. | 46,XX pat transmission of both X | mild MR, short stature, hypotonia | 11950856 |
| UPD(Xq13.1→qter)  Pat | **85.05** | 2y | n.a. | congenital abnormalities of face and neck | 34284807 |
