# Supplementary figures and images for: Uniparental disomy: expanding the clinical and molecular phenotypes of whole chromosomes
Source: Front Genet. 2023 Oct 4;14:1232059. doi: 10.3389/fgene.2023.1232059 (PMC10582337; doi:10.3389/fgene.2023.1232059)

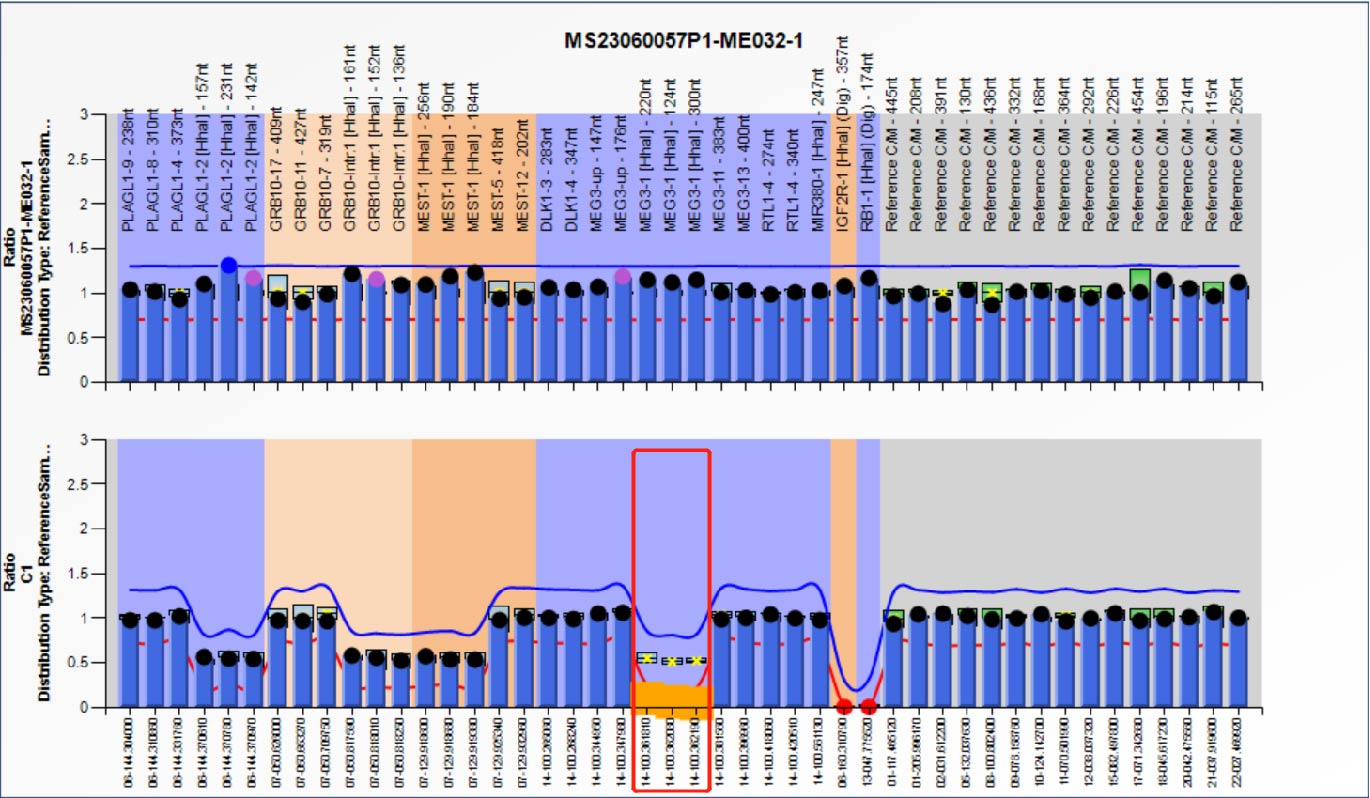

Supplement: Supplementary file 2 [file Image1.JPEG]
