# Supplementary material for: Uniparental disomy: expanding the clinical and molecular phenotypes of whole chromosomes
Source: Front Genet. 2023 Oct 4;14:1232059. doi: 10.3389/fgene.2023.1232059 (PMC10582337; doi:10.3389/fgene.2023.1232059)
Supplement: Supplementary file 3 [file DataSheet2.docx]

**Supplementary Table 2. Summary of the documented pathogenic homozygous variants of UPD(2), (9) and (14).**

| **Gene** | **Locus**  **(mutation)** | **Origin** | **Clinical Phenotype** | **Diseases/syndrome** | **PMID** |
| --- | --- | --- | --- | --- | --- |
| SRD5A2 | 2p23.1  c.591G>T,  E197D | Pat | pseudohermaphroditism | Pseudohermaphroditism | 10999800 |
| LRP2 | 2q31.1  c.11469_11472delTTTG (p.Cys3823TrpfsX159) | Pat | hypertelorism, bilateral coloboma, absence of the corpus callosum, malrotation of the gut, bilateral inguinal hernias | Donnai-Barrow syndrome | 18553518 |
| DYSF | 2p13  c.1471dupA  (p.M491Nfs*15) | Pat | hyperCKemia and mild muscle weakness | Dysferlinopathies | 36464789 |
| UGT1A1 | 2q37.1  c.170_171A(TA)6TAA | Pat | non-hematologic jaundice | Crigler-Najjar syndrome type 1 (CNs1) | 35199468 |
| LCHAD | 2p23.3  c.1526G>C,  (p.Glu510Gln) | Pat | abnormal acylcarnitine pattern | Long-chain 3-hydroxyacyl-CoA dehydrogenase deficiency (LCHADD) | - 20583174 |
| MERTK | 2q14.1  IVS10-2 A>G, | Pat | retinal degeneration | Second retinal dystrophy | 11727200 |
| CYP1B1 | 2p22.2  Heterozygous deletion | Pat | / | Primary Congenital glaucoma(PCG) | 31251480 |
| RAB3GAP1 | 2q21.3  c.665delC (p.Pro222HisfsTer30) | Pat | Spasticity, global neurodevelopmental delay,  hypotonia, microcephaly, ptosis, bilateral congenital cataracts, microphthalmia,  microcornea, strabismus | Warburg micro syndrome (WARBM) | 32599602 |
| CNGA3 | 2q11.2  c.778G>C;  p.(D260H) | Pat | / | Achromatopsia (ACHM) | 34360608 |
| ABCB11 | 2q31.1  c.2492C>T,  p.R832C | Pat | hepatocellular cholestasis, pruritus, elevated serum bile acid concentrations, exclusion of other causes of cholestasis | Familial intrahepatic cholestases (FICs) | 26678486 |
| CHRND | 2q37.1  c.236T>A  (p. Ile79Lys); c.340G>C  (p.Val114Leu | Pat | arthrogryposis, polyhydramnios and  absent stomach bubble in the fetus; (after birth)  significant joint contracture, small chest and mild dysmorphic  facial features, including micrognathia. | Lethal multiple pterygium syndrome | 29399782 |
| SMARCAL1 | 2q35  Frameshift | Pat | spondyloepiphyseal dysplasia and nephropathy | Schimke immuno-osseous dysplasia (SIOD) | 34031513 |
| GGCX | 2p11.2  c. 44-1G>A | Pat | / | Vitamin K-dependent coagulant factor deficiency (VKCFD) | 27681307 |
| NEB | 2q23.2  c.24681C>G,  p. (Tyr8227*) | Pat | hypotonia, weakness, and delayed motor development | Nemaline myopathy 2 | 29656286 |
| ABCA12 | 2q34  exon7, c.859C>T,  p. R287X | Pat | abnormal desquamation and extreme skin thickening and hardening over the entire body;  severe growth delay and oligohydramnios in prenatal | Harlequin ichthyosis (HI) | 19664001 |
| DGUOK | 2p13.1  C.679G>A | Pat | Jaundice, coagulopathy, severe hypoglycemia, severe liver damage | Mitochondrial DNA depletion syndrome (MDS) | 32482602 |
| ANTXR1 | 2p13.3  c.1435-2A>T | Pat | short stature, facial dysmorphic features including macrocephaly,  coarse face, saddle nose as well as wide nasal bridge | GAPO syndrome(growth retardation, alopecia, pseudoanodontia and optic atrophy) | 31425299 |
| FOXE1 | 9q22,  c.412T>C;  F137S | Mat | severe thyroid hypoplasia with normal IQ, short stature, small cardiac ventricular septal defect, hip subluxation, dysmorphic facial features, persistent velopharyngeal incompetence | Syndromic Congenital Hypothyroidism | 20484477 |
| RMRP | 9p13.3 | Mat | impaired skeletal growth and cellular immunity | Cartilage-hair hypoplasia (CHH) | 9156319 |
| SURF1 | 9q34  c.751C>T | Mat | Slightly delayed motor development at 18 month, psychomotor development arrest with ataxia, loss of walking and dysarthria at 2-years-old, dystrophy, microcephaly, severe muscular hypotonia and truncal ataxia at 3-years-old. | Leigh syndrome (LS) | 16773507 |
| SARDH | 9q34  c.211G>T | Mat | Developmental motor and cognitive delay, abnormal movements | Sarcosinemia | 22825317 |
| MLLT3, LAMC3,HNRNPU, SLC6A8 | / | Mat | developmental delay and intellectual disability, congenital heart defect | / | 24356988 |
| IL11RA | 9p13.3  c.707T>C (p.Leu236pro) | Mat | broad and horizontal eyebrows, long philtrum, multiple craniosynostosis | Craniosynostosis and Tooth abnormalities | 32277509 |
| B4GALT1 | 9p21.1,  c.579C>G  (p.Tyr193Ter) | Mat | facial dysmorphism, hypotonia, decreased coagulation factors and increased serum transaminase, skin lesions, bone abnormalities, growth retardation, hypothyroidism | IId type congenital disorders of glycosylation | 30653653 |
| SURF1 | 9q34.2  c.241-1G>C | Pat | Low birth weight, thrive failure, psychomotor regression, mildly delayed development, hydrocephalus with seizures and vomiting, increased lactate, atrial septal defect | Leigh syndrome | 31454184 |
| AUH | 9q22.31  c.373C>T  p.(Arg125Trp) | Unknown | organic aciduria and elevated C5OH | 3-methylglutaconic aciduria, type I | 23100014 |
| ASS1 | 9q34.11,  c.571G>A  p.(Glu191Lys) | Unknown | Elevated citrullin, spastic quadriparesis, developmental delay  (9q21.11q21.33 duplication and 9q21.33q34.3 ROH) | Citrullinemia | 25118026 |
| POMT2 | 14q24.3  c.1502A>C  (p.E501A) | Mat | muscular dystrophy(proximal muscle atrophy, generalized hypotonia, progressive contractures), low left ventricular ejection and mild restrictive lung disease | Walker-Warburg syndrome and Muscle-Eye-Brain disease | 29759639 |
| GALC | 14q31.3  c.1394C>T,  （p.Thr465Ile） | Pat/Mat  het; (consanguineous) | refractory seizures, growth failure, cognitive impairment (could not follow objects, no head control, unable to recognize his mother) | Krabbe disease | 30209698 |
| RDH12 | 14q24.1  c.437T>A  (p.Val146Asp)、c.184C>T  (p.Arg62Ter)  c.524C>T  (p.Ser175Leu) | Pat/Mat  het; | retinitis pigmentosa; leber congenital amaurosis; early onset of severe retinal dystrophy; cone rod dystrophy | Inherited eye disease | 35006499 |
| NPC2 | 14q24.3  c.358C>T  (Pro120Ser) | Pat/Mat,  het; | hepatosplenomegaly and progressive neurological deterioration | Niemann-Pick C disease | 23791309 |
| LTBP2 | 14q24.3  c.5446dupC | Pat/Mat,  het; | / | Microspherophakia | 20617341 |
| ESRRB | 14q24.3  c.1018_1024 dupGAGTTTG  (p.V342GfsX44) | Pat/Mat,  het; | sensorineural hearing loss | nonsyndromic hearing impairment | 18179891 |
| VIPAS39 | 14q24.3  missense c.1130G > C  (Arg377Pro)) | Pat/Mat,  het; | arthrogryposis, renal tubular dysfunction and cholestasis; (2.5 month old infant) dysmorphic features, including small anterior fontanel, low set ears, beaked nose and high arched palate, unilateral choanal atresia, club foot, bilateral developmental dislocation of hip | ARC syndrome | 26808426 |
