# Supplementary material for: Uniparental disomy: expanding the clinical and molecular phenotypes of whole chromosomes
Source: Front Genet. 2023 Oct 4;14:1232059. doi: 10.3389/fgene.2023.1232059 (PMC10582337; doi:10.3389/fgene.2023.1232059)
Supplement: Supplementary file 4 [file DataSheet1.docx]

**Supplementary Table 1**. Summary of heterozygous variants analyzed by the trio-WES analysis in the UPD(14).

| **Gene** | **Locus** | **Variants** | **Proband** | **pat** | **mat** | **OMIM phenotype/Inheritance** |
| --- | --- | --- | --- | --- | --- | --- |
| NEB | 2q23; 2:152350704  Exon175;NM_001271208 | c.24654_  24655del, p.  R8218Sfs*9 | Het | / | Het | Nemaline myopathy (AR) |
| CC2D2A | 4p15; 4:15603037  Exon38;NM_001080522 | c.4852C>T,  p.R1618C | Het | Het | / | Coach syndrome (AR)、Meckel syndrome 6 (AR)、Joubert syndrome type 9 (AR) |
| CCDC114 | 19q13; 19:48821643  Intron3;NM_144577 | c.249+1G>T,  p.(?) | Het | Het | / | Primary ciliary dyskinesia type 20 (AR) |
| POLRMT | 19p13; 19:624843  Exon5;NM_005035 | c.1016T>C,  p.L339P | Het | / | Het | Combined oxidative phosphorylation deficiency type 55 (AD or AR) |
| RTL3 | Xq21; X:77913761  Exon2;NM_152694 | c.157C>T, p.  R53X | Het | / | Het | Developmental disorders (unknown genetic pattern) |
| SMPD1 | 11p15; 11:6411930  Exon1;NM_000543 | c.102_  103ins6, p.  L35_V36insAL | 20% mosaic ? | / | Het | Niemann-Pick disease A (AR)、Niemann-Pick disease B (AR) |
| TRIP11 | 14q32; 14:92469878  Exon11;NM_004239 | c.4442C>T,  p.A1481V | Het | / | Het | Osteochondroplasia (AR), Chondrodysplasia type 1A (AR) |
| TRPV6 | 7q34; 7:142572878  Exon9;NM_018646 | c.1282G>A,  p.G428R | Het | Het | / | Neonatal temporary hyperparathyroidism (AR) |
| AMT | 3p21; 3:49456724  Exon6;NM_000481 | c.665G>A, p.  R222H | Het | / | Het | Glycine encephalopathy (AR) |
